# Frequency and Associated Risk Factors of Hepatitis B Virus and Hepatitis C Virus Infections in Children at a Hepatitis Prevention and Treatment Clinic in Lahore, Pakistan

**DOI:** 10.7759/cureus.7926

**Published:** 2020-05-02

**Authors:** Iqtadar Seerat, Humaira Mushtaq, Memona Rafiq, Abdul Nadir

**Affiliations:** 1 Pediatric Gastroenterology & Hepatology, Pakistan Kidney and Liver Institute and Research Center, Lahore, PAK; 2 Pediatric Gastroenterology & Hepatology, Pakistan Kidney and Liver Institute & Research Centre, Lahore, PAK; 3 Gastroenterology & Hepatology, Pakistan Kidney and Liver Institute & Research Centre, Lahore, PAK

**Keywords:** children, hepatitis c virus, hepatitis b virus, frequency, risk factors

## Abstract

Objective

This study evaluated the frequency of hepatitis B virus (HBV) and hepatitis C virus (HCV) infection and the associated horizontal risk factors in children being screened for viral hepatitis in Lahore, Pakistan.

Methods

Children aged 15 years or younger who were brought to a specialized outpatient viral hepatitis clinic affiliated with a tertiary hospital in Lahore, Pakistan, for viral hepatitis screening from March 2017 to March 2018 were enrolled. Children were screened for HBV and HCV infection by enzyme-linked immunosorbent assay; if results were positive, HBV and HCV concentrations were quantitatively assayed by polymerase chain reaction. Children positive for HBV or HCV infection were matched with 100 controls of the same age and sex. All subjects completed a questionnaire on viral infection and its associated risk factors.

Results

During the study period, 3500 children living in the Punjab Province of Pakistan were screened for HBV and HCV infection. Of these children, 28 (0.8%) were positive for HBV and 66 (1.88%) were positive for HCV. A comparison of the 94 (2.68%) children positive for HBV or HCV with 100 controls identified several risk factors associated with infection. Unexpectedly, ten (35.7%) of the 28 HBV-positive children were born of HBV-negative mothers and had been fully vaccinated for HBV during infancy.

Conclusion

The frequency of HCV infection was higher than that of HBV infection among Pakistani children aged ≤15 years. Several horizontal risk factors were found to cause viral hepatitis. Several children born of HBV-negative mothers and vaccinated for HBV during infancy later developed HBV infection.

## Introduction

Hepatitis B virus (HBV) and hepatitis C (HCV) virus are the most common viruses resulting in chronic liver disease (CLD) [1]. Of the approximately 170 million people worldwide infected with HCV, 71 million progress to have CLD, putting them at risk of cirrhosis and hepatocellular carcinoma (HCC) [2-3]. HCV-associated CLD remains a major indication for liver transplantation [4].

Almost 360 million people worldwide, or approximately 6% of the global population, are chronically infected with HBV [5]. The incidence of HBV infection has decreased after the introduction of international vaccination schemes and meticulous screening of people donating blood. However, large numbers of children are infected and will require long-term care in outpatient clinics. Despite the generally benign nature of chronic hepatitis B in children, studies have estimated that 1% to 5% develop cirrhosis and 2% to 5% develop HCC during childhood [6].

Horizontal transmission, especially in children, remains an important cause of HBV and HCV infection in developing countries, including Pakistan. Both viruses are blood-borne and spread by unsafe blood transfusion and the use of non-sterilized syringes and surgical equipment. Few studies to date, however, have assessed the incidence of HBV and HCV infection in children. The prevalence of HCV infection in the Punjab Province of Pakistan remains very high. The present study, therefore, assessed the frequency and risk factors for viral hepatitis in Punjabi children being screened at a specialized urban viral hepatitis clinic affiliated with the Pakistan Kidney and Liver Institute and Research Center, a tertiary hospital in Lahore, Pakistan.

## Materials and methods

**Figure 1:**
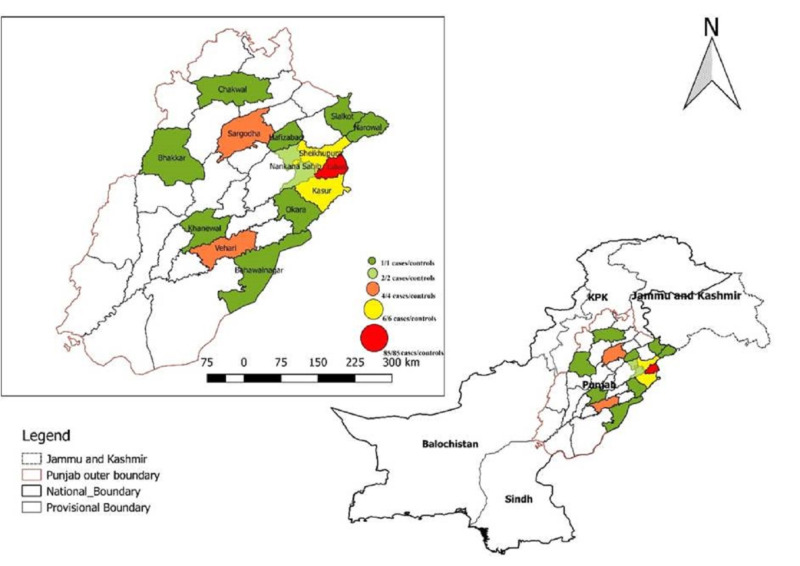
Geographical distribution of viral and non-viral hepatitis in children screened at HPTC, Lahore, Pakistan HPTC, Hepatitis Prevention and Treatment Clinic

Children aged ≤15 years living in Punjab Province, the second largest province in Pakistan, screened for viral hepatitis at the Hepatitis Prevention and Treatment Clinic (HPTC) in Lahore, Pakistan, from March 2017 to March 2018 were recruited. Most subjects lived in Lahore, the largest city in Punjab Province, but others lived in various geographic regions throughout this province (Figure 1).

All children were otherwise healthy and were screened as a precautionary measure or due to a family member being infected with HBV or HCV.

Blood was drawn from each subject, and serum was prepared. Subjects were initially tested for HBV and HCV infection by enzyme-linked immunosorbent assays (ELISA) for hepatitis B surface antigen (HBsAg) and anti-HCV antibody. If ELISA results were positive, HBV and HCV concentrations were quantitatively determined by polymerase chain reaction. Children positive for HBV or HCV infection were matched 1:1 with control, randomly selected children of the same age and sex who had not had any significant medical conditions.

All subjects completed a questionnaire on viral incidence and associated risk factors, as well as questions evaluating maternal HBV and HCV infection. Data were analyzed using IBM SPSS Statistics for Windows, Version 22.0. (Armonk, NY: IBM Corp.). Associations between dependent and independent variables were assessed by chi-square tests. Data normality was assessed using the Kolmogorov test and regression analysis. The p-values of less than 0.05 were considered statistically significant. Multivariable logistic regression analysis was performed, with results presented as odds ratios (ORs) and 95% confidence intervals (CIs).

## Results

This comparative, cross-sectional study analyzed the frequency of HBV and HCV infection and risk factors associated with infection in Punjabi children aged ≤15 years. Of the 3500 children screened, 94 (2.68%) were positive for HBV and HCV, including 28 (0.8%) positive for HBV and 66 (1.88%) positive for HCV. The mean age of these 94 infected subjects was 10.1 ± 3.9 years, whereas the mean age of the 100 matched controls was 9.6 ± 3.7 years. Of the 94 positive subjects, 13 (13.8%) were younger than age 5, 29 (30.9%) were aged five to 10 years, and 52 (55.3%) were aged 10 to 15 years. Of the 100 control subjects, eight (8%) were younger than age five, 41 (41%) were aged five to 10 years, and 51 (51%) were aged 10 to 15 years.

Of the subjects assessed, 4% of girls and 10% of boys were HBV-infected, whereas 12.5% of girls and 20.5% of boys were HCV-infected. We found that 89% of HBV-infected children and 79% of HCV-infected children had a family history of infection. Univariable analysis showed that a family history of HBV infection and surgical procedures in government hospitals were significant risk factors for HBV infection (P-value <0.001 each). Receipt of blood transfusions, exposure to dental and surgical procedures at public hospitals, and a family history of hepatitis were significantly associated with HCV infection (P-values <0.05 each). Variables with P-values <0.20 were included in the multivariable logistic model. Multivariable logistic regression analysis identified two risk factors significantly associated risk with HBV infection: having an HBV-infected mother and circumcision by a barber (Table 1).

**Table 1:** Multivariable logistic regression of risk factors in matched actual case/control groups of children screened for HBV HBV, Hepatitis B virus; HCV, Hepatitis C virus

Variable	Status	Cases/Controls	Odds Ratio	95% Confidence Interval	P-value
Vaccination Status	Yes	24/26	0.46	0.07-2.75	0.39
	No	4/2	-		
Toothbrush Sharing	Yes	2/15	0.90	0.6-1.2	0.49
	No	26/13	-	-	
Mother HBV Status	Positive	5/1	5.8	0.3-53	0.11
	Negative	23/27	-	-	
Maternal HCV Status	Positive	12/11	1.15	0.39-3.3	0.78
	Negative	16/17	-	-	
Circumcision	Home	18/10	1.53	0.3-1.81	0.003
	Hospital	0/4	-	-	
	N/A	10/14	-	-	

Multivariable logistic regression analysis identified several risk factors independently associated with HCV infection: male sex, receipt of intravenous injections, hospital admissions, exposure to surgical and dental procedures, circumcision by barbers, and having an HCV-infected mother (Table 2). 

**Table 2:** Multivariable logistic regression for risk factors in matched actual case/control groups of children screened for HCV HCV, Hepatitis C virus

Variable	Status	Cases/Controls	Odds Ratio	95% Confidence Interval	P-value
Gender	Male	43/31	1.14	0.8-2.3	0.019
	Female	23/35	-	-	
Intravenous Injection	<5 injections	11/14	-	-	0.53
	>5 injections	55/52	1.3	0.5-2.9	
Exposure To Dental Procedure	Yes	10/4	1.10	0.7-1.5	0.57
	No	56/62	-	-	
Hospitalization Status	Yes	26/13	1.33	0.8-2.0	0.01
	No	40/53	-	-	
Exposure To Surgical Procedure	Yes	10/2	1.14	0.7-1.6	0.04
	No	56/64	-	-	
Exposure To Head Shave/Trim	Yes	23/32	0.78	0.4-1.2	0.30
	No	43/34	-	-	
Toothbrush Sharing	Yes	6/9	0.94	0.6-1.3	0.78
	No	60/57	-	-	
Maternal HCV Status	Positive	34/30	1.12	0.6-1.8	0.62
	Negative	32/36	-	-	
Circumcision	Home	38/20	1.51	0.2-0.8	0.01
	Hospital	0/11	-	-	
	N/A	28/35	-	-	

Of the 28 children positive for HBV cases, 10 (35.7%) were born of HBV-negative mothers and received three doses of HBV vaccine as infants.

## Discussion

HCV occurs in about 0.15% of children aged six to 11 years and 0.4% of those aged 12 to 19 years. A previous study performed by the Pakistan Medical and Research Council (PMRC) from mid-2007 to mid-2008 reported the prevalence of HBV and HCV in hospitalized children and those in outpatient clinics [7]. That study found that the incidence of infection varied in different parts of the country, with most infected patients being from Baluchistan and Sindh Provinces. Only 2.5% of recruited patients were positive for HBsAg, whereas 4.9% were positive for anti-HCV antibody, making the overall infection rate 7.4% [8]. Another study, involving 3533 Pakistani children, found that 65 (1.8%) were positive for HBsAg and 55 (1.6%) positive for anti-HCV antibody, making the overall infection rate 3.3% [9]. The main risk factor for HBV infection was the receipt of therapeutic injections. Another Pakistani study reported that the prevalence of HBsAg in pediatric populations was 2.4% (range 1.7-5.5%), whereas the prevalence of anti-HCV antibody was 2.1% (range 0.4-5.4%) [10]. The major risk factors for the transmission of HBV and HCV included the use of contaminated needles during medical care, drug abuse, and unsafe transfusion of blood and blood products.

A study in Bangladesh reported that the risk factors for HBV infection in children were surgical procedures, intravenous, and intramuscular injections, mothers positive for HBV infection, and unhygienic haircuts by barbers [11]. The risk factors for HCV transmission in Egyptian children included a positive family history of HCV infection, parenteral injections, blood transfusion, surgery, and hospitalization [12].

In comparison, our study of 3500 children showed that the frequency of combined HBV and HCV infection was 2.68%, including 0.8% infected with HBV and 1.88% infected with HCV. Risk factors significantly associated with HCV infection included male sex, intravenous injections, exposure to surgical operations and dental procedures, multiple hospitalizations, HCV-positive mothers, and circumcision by barbers. The significant risk factors associated with HBV infection were male sex, HBV-positive mothers, and circumcision by barbers.

Steps to curtail unwanted injections and the use of contaminated needles should be strongly encouraged. The proper sterilization surgical instruments and apparatus used in body piercing, along with pre-transfusion screening tests, are essential to avoid virus transmission [13-14]. A mass immunization policy may prevent HBV infection in Pakistani children. This policy should include the vaccination of infants aged 6 weeks, 10 weeks, and 14 weeks.

We found that 10 (35.7%) of the 28 HBV-positive children were born of HBV-negative mothers and had been fully vaccinated for HBV during infancy. These children did not have any other active health issues, suggesting that a faulty immunization technique or improper storage of vaccines may have led to HBV infection, despite their mothers being HBV-negative and receiving a vaccination themselves.

Due to a lack of resources, we were unable to measure the titer of the anti-hepatitis B surface (Anti-HBS) antibody or assess maternal infection. Fully vaccinated children with low antibody levels may require a booster dose if the level remains low despite being fully vaccinated. Administration of vaccine and hepatitis B immunoglobulin promptly after birth may enhance immune success rates to 90% and 98% in Hepatitis B e-Antigen (HBeAg)-positive and negative mothers, respectively [15-17].

The likelihood of maternal HCV transfer to a newborn is <5% [18-20]. However, it was not possible to determine the frequency of vertical transmission of the virus to the 66 HCV-positive children in this study because maternal HCV status was not known, and laboratory tests were not performed during early infancy.

## Conclusions

The frequency of HCV infection is higher than that of HBV infection in Pakistani children, with viral hepatitis being due in part to unsafe medical practices. Some children of HBV-negative mothers who were vaccinated as infants were positive for HBV. National policies should be aligned with global recommendations.
